# The effects of mechanical insufflation-exsufflation on lung function and complications in cardiac surgery patients: a pilot study

**DOI:** 10.1186/s13019-021-01738-x

**Published:** 2021-12-09

**Authors:** Meng-Fang Wu, Tsai-Yu Wang, Da-Shen Chen, Hsiu-Fong Hsiao, Han-Chuang Hu, Fu-Tsai Chung, Ting-Yu Lin, Shu-Min Lin

**Affiliations:** 1 Department of Respiratory Therapy, School of Medicine, Chang Gung Memorial Hospital, Chang Gung University, Taipei, Taiwan; 2 Department of Thoracic Medicine, Chang Gung Memorial Hospital, Chang Gung University, 199 Tun-Hwa N. Rd, Taipei, Taiwan

**Keywords:** Physiotherapy, Cardiac surgery, Complications, Lung functions, Mechanical insufflation-exsufflation, Atelectasis

## Abstract

**Background:**

Postoperative positive pressure lung expansion is associated with decreased pulmonary complications and improved clinical outcomes. The aim of the present study was to compare the differences in post-operative pulmonary complications and clinical outcomes between two groups of study subjects who underwent cardiac surgery; one included subjects who received mechanical insufflation-exsufflation (MI-E) and the other included subjects who received intermittent positive pressure breathing (IPPB) therapy.

**Methods:**

This retrospective study included 51 subjects, who underwent cardiac surgery in an intensive care unit of a tertiary hospital during the time period from June 2017 to February 2018. After liberation from mechanical ventilation, the subjects received lung expansion therapy by means of two types of positive pressure devices, MI-E (n = 21) or IPPB (n = 30). The pulmonary complications, lung function, and clinical outcomes were compared between the two groups.

**Results:**

Subjects in both groups displayed similar baseline characteristics and underwent similar types of surgical procedures. Compared to subjects who received non-oscillatory therapy, those who received MI-E therapy had higher post-operative force vital capacity (58.4 ± 4.74% vs. 46.0 ± 3.70%, *p* = 0.042), forced expiratory volume in one second (62.4 ± 5.23% vs. 46.8 ± 3.83%, *p* = 0.017), and peak flow rate (67.1 ± 5.53 L vs. 55.7 ± 4.44 L *p* = 0.111). However, the incidence of chest pain was higher in the MI-E group (n = 13, 61.9%) than in the IPPB group (n = 4, 16.7%; odds ratio, 0.123, 95% confidence interval, 0.03–0.45; *p* = 0.002). The length of hospital and ICU stay, development of atelectasis, pneumonia, and pleural effusion were similar in both the groups.

**Conclusion:**

Both IPPB and MI-E therapies have similar effects on preventing post-operative complications in cardiac surgery patients. However, compared to IPPB therapy, MI-E therapy was associated with better-preserved pulmonary function and higher incidence of chest pain.

**Supplementary Information:**

The online version contains supplementary material available at 10.1186/s13019-021-01738-x.

## Background

Literature reveals that the incidence of postoperative pulmonary complications (PPCs), subsequent to in cardiac surgery, ranges from 1.9% to 7.9% [1, 2], which include prolonged hospital stay or postoperative morbidity and mortality [3]. Recently, the European joint taskforce guidelines for perioperative clinical outcome (EPCO) considered respiratory infection, respiratory failure, pleural effusion, atelectasis, pneumothorax, bronchospasm and aspiration pneumonitis as the composite measures of PPCs [4]. Changes to the respiratory system occur immediately on induction of general anesthesia: respiratory drive and muscle function are altered, lung volume reduced, and atelectasis developed in majority of patients receiving a neuromuscular blocking agent. Post-operative sputum retention is a common phenomenon. General anesthesia, especially with a tracheal tube, causes impairment of the mucociliary transport in the airways and this effect may persist during the postoperative period. This combination of reduced FRC, residual atelectasis, ineffective cough and abnormal respiratory control generates an ideal situation for the development of PPCs [5].

Although evidence that supports the use of respiratory physiotherapy in hospitalized patients is controversial [6], this type of intervention is widely used in clinical practice [7]. A wide range of techniques of respiratory physiotherapy are available, in order to assist lung expansion and facilitate the clearance of pulmonary secretions. A variety of therapeutic interventions can be performed using the physical abilities of the physiotherapist and/or the patient, such as deep breathing exercises, manual pulmonary clearance techniques and forced expiration techniques [8]. Other types of therapeutic interventions, such as breathing exercises with positive pressure, require specific devices. Several treatment modalities, such as prophylactic continuous positive airway pressure ventilation (CPAP) have been shown to reduce PPCs [9]. A randomized controlled trial reported that supplementary therapy using bilevel positive airway pressure (BiPAP), in addition to the protocols, significantly reduced the incidence of atelectasis [10]. Moreover, intermittent positive pressure breathing (IPPB) is also a popular method for the prevention of atelectasis after cardiac surgery. The primary purpose of using positive pressure devices for breathing exercises is to facilitate lung expansion and thus prevent the development of atelectasis and to improve the clearance of secretions.

Cough plays a critical role in airway clearance. Cough augmentation techniques, including lung volume recruitment or mechanically assisted cough, are used to prevent respiratory complications associated with muscle weakness [11]. Mechanical insufflation-exsufflation (MI-E) consists of lung insufflation by means of positive pressure, followed by an active negative-pressure exsufflation, which simulates a cough and moves the pulmonary secretions towards the mouth [11]. MI-E is generally used in patients with neuromuscular diseases or muscle weakness, caused by injuries to the central nervous system [12]. Moreover, MI-E has also been used to facilitate airway mucus clearance in mechanically ventilated ICU patients [13]. Considering the theoretical benefits of providing positive pressure for lung expansion and facilitation of sputum clearance through cough augmentation, it can be safely suggested that post-operative use of MI-E in cardiac surgery patients can aid in the prevention of PPCs. Therefore, the aim of the present study was to compare the effects of MI-E with that of IPPB, pertaining to lung expansion and subsequent prevention of lung atelectasis, after cardiac surgery.

## Material and methods

### Study population

This retrospective study included subjects, who underwent cardiac surgery in Chang Gung Memorial Hospital, a tertiary hospital in Taiwan, during the time period from June 2017 to February 2018. Subjects who had a history of previous thoracic surgery, thoracic abnormalities and subjects younger than 18 years of age were excluded from the study. Initial survey recruited 82 patients received cardiac surgery. Among them, 15 patients were excluded due to received postoperative mechanical ventilation < 24 h, 10 patients were excluded due to failure to perform postoperative spirometer, 3 patients were excluded due to received physiotherapy < 3 days, and 3 patients were excluded due to absence of post-operative chest X ray study. Thus, there were 51 patients included in the final analysis. The Chang Gung Medical Foundation Institutional Review Board approved the study and waived the requirement for informed consent, owing to the retrospective nature of this study.

### Study design

The medical records of all the subjects included in the current study were reviewed, in order to extract data regarding the clinical characteristics and laboratory results. Additionally, information regarding the baseline characteristics, comorbidities, pulmonary function tests, the type of cardiac surgery performed, the type of IPPB used (IPPB or oscillatory IPPB); the duration of ventilator use; the duration of ICU stay and hospital stay and the type of PPCs encountered was collected.

One day prior to the surgery, all subjects were given instructions regarding breathing techniques such as deep diaphragmatic ventilation and effective coughing, by two respiratory therapists. Postoperatively, all the subjects received mechanical ventilation (pressure-controlled, peak airway pressure of 25 cm H_2_O, targeting a tidal volume of 8 mL/kg, an initial positive end-expiratory pressure (PEEP) of 10 cm H_2_O, with a fraction of inspired oxygen (FiO_2_) level designed to keep arterial oxygen saturation at > 95%). Weaning was initiated if the subjects were normothermic and hemodynamically stable, and operative revision was not required. Weaning was facilitated by reducing the respiratory rate and changing to pressure support ventilation if spontaneous breathing had resumed. Subjects were extubated if they were fully conscious, responsive to commands, had sufficient protective airway reflexes and met the following criteria: partial pressure of oxygen > 70 mm Hg at an FiO_2_ of 0.35 and a PEEP of 7 cm H_2_O; and partial pressure of carbon dioxide < 46 mm Hg at a pressure support of < 8 cm H_2_O. On extubation, two respiratory therapists guided the implementation of either IPPB (ResMed's Astral 150) or MI-E (Philips E70® Cough Assistor) therapy. The selection of either IPPB or MI-E was dependent on the availability of devices in the ICU. In all the subjects, the peak airway pressure, ranging from 10 to 25 cm H_2_O, was adjusted during each session, resulting in widening of the thoracic cage. The simultaneous attainment of deep inspiration at the peak pressure confirmed the satisfactory effect of respiratory therapy. The target of respiratory therapy was to attain more than ten satisfactory effects during each session.

All the subjects underwent pulmonary function tests (PFT) prior to the surgery. Moreover, after performing either IPPB or MI-E therapy on all the subjects for five days, pulmonary function tests were repeated while patients under adequate pain control as the routines of CVS ICU. Two measurements of PFT were taken and the better result was recorded. Two independent pulmonologists, blinded to the group allocation in the study, analyzed postero-anterior (PA) chest X-ray films of subjects, which were taken before surgery and thereafter daily up to the fifth postoperative day. Atelectasis, lobar collapse, pulmonary infiltrations, and pleural effusions were recorded.

### Definitions

The definitions of PPCs, used in the current study, were in accordance with the European perioperative clinical outcome (EPCO) [5]. In brief, the definition of atelectasis was lung opacification with mediastinal shift, hilum or hemidiaphragm shift towards the affected area, with compensatory hyperinflation in the adjacent non-atelectatic lung. Pleural effusion was defined as CXR with the radiographic feature of blunting of the costophrenic angle, loss of sharp silhouette of the ipsilateral hemidiaphragm in upright position, displacement of adjacent anatomical structures, or (in supine position) hazy opacity in one hemithorax with preserved vascular shadows. The diagnostic criteria for pneumonia was CXR with at least one of the following radiographic features: infiltrate, consolidation, cavitation; plus at least one of the following clinical features: fever > 38 °C with no other evident cause, white cell count < 4 or > 12*10^9^ L^−1^, subjects of age greater than 70 years with altered mental status with no other evident cause; plus at least two of the following features: new purulent/ changed sputum, increased secretions/suctioning, new/worse cough/dyspnea/tachypnea, rales/bronchial breath sounds, worsening gas exchange.

### Statistical analysis

Data were expressed as mean ± SEM (standard error of the mean). Two-tailed Student’s t test was used to compare the continuous variables in the two groups, while Mann–Whitney test was used for the non-normal distributions. Categorical variables were compared by means of the **x**^2^ or Fisher’s exact tests. P value of less than 0.05 was considered to be statistically significant. Analysis was carried out using SPSS (version 13.0; SPSS; Chicago, IL) statistical software.

## Results

### Demographics and clinical characteristics of subjects

The current study involved a total of 51 subjects, who underwent cardiac surgery and received either IPPB or MI-E therapy in our hospital, during the time period from June 2017 to February 2018. 21 (41.2%) of the 51 subjects received oscillatory IPPB (MI-E therapy). The baseline demographics and clinical characteristics of the study subjects are listed in Table 1. The study subjects were divided into two groups based on the type of respiratory therapy received: the MI-E group and the IPPB group. The mean age of the subjects in the MI-E and IPPB groups were 63.8 years and 62.5 years, respectively. The proportion of male subjects in the MI-E and IPPB groups were 66.7% and 56.7%, respectively. Other characteristics including BMI, habit of smoking, type of surgery, maximal inspiratory pressure, duration of ventilator use and the duration of ICU stay were similar between the two groups. The levels of white blood cell, sodium, potassium, and creatinine were measured on day 1 after operation.

**Table 1:** Baseline characteristics of the study subjects

Variables	MI-E group, n = 21	IPPB group, n = 30	*p* value
Age, years	63.8 ± 2.5	62.5 ± 2.3	.715
Male gender, n (%)	14 (66.7)	17 (56.7)	.472
BMI, kg/m^2^	24.0 ± 0.8	24.7 ± 0.7	.518
Smoker, n (%)	10 (47.6)	15 (50)	.615
DM, n (%)	10 (47.6)	11 (38.1)	.917
HTN, n (%)	10 (47.6)	17 (56.7)	.524
CAD, n (%)	9 (42.9)	15 (50.0)	.615
COPD, n (%)	1 (4.8)	1 (3.3)	.796
Asthma, n (%)	0 (0)	1 (3.3)	.999
Chronic renal disease, n (%)	9 (42.9)	12 (40.0)	.838
Types of surgery			.933
Thoracic endovascular aortic repair	3 (14.3)	3 (10.0)	
Valve replacement surgery	10 (47.6)	16 (53.3)	
Coronary artery bypass grafting	8 (38.1)	10 (33.3)	
Septal repair surgery	0 (0)	1 (3.3)	
White Blood Cell count	9.7 ± 1.2	11.2 ± 1.0	.333
Creatinine	1.4 ± 0.3	1.9 ± 0.4	.407
Sodium	140.0 ± 0.8	139.3 ± 0.8	.537
Potassium	3.9 ± 0.1	4.0 ± 0.1	.563
Maximal inspiratory pressure, (cm H_2_O)	36.2 ± 1.9	37.4 ± 1.7	.644
Duration of ventilator use (days)	2.1 ± 0.4	2.7 ± 0.6	.453
Length of ICU stay (days)	4.2 ± 0.6	4.8 ± 0.8	.560

MI-E: Mechanical insufflation-exsufflation, IPPB: Intermittent positive pressure breathing, BMI: Body mass index, ICU: Intensive care unit

### Comparison of post-operative changes in lung function between the two groups

**Fig. 1:**
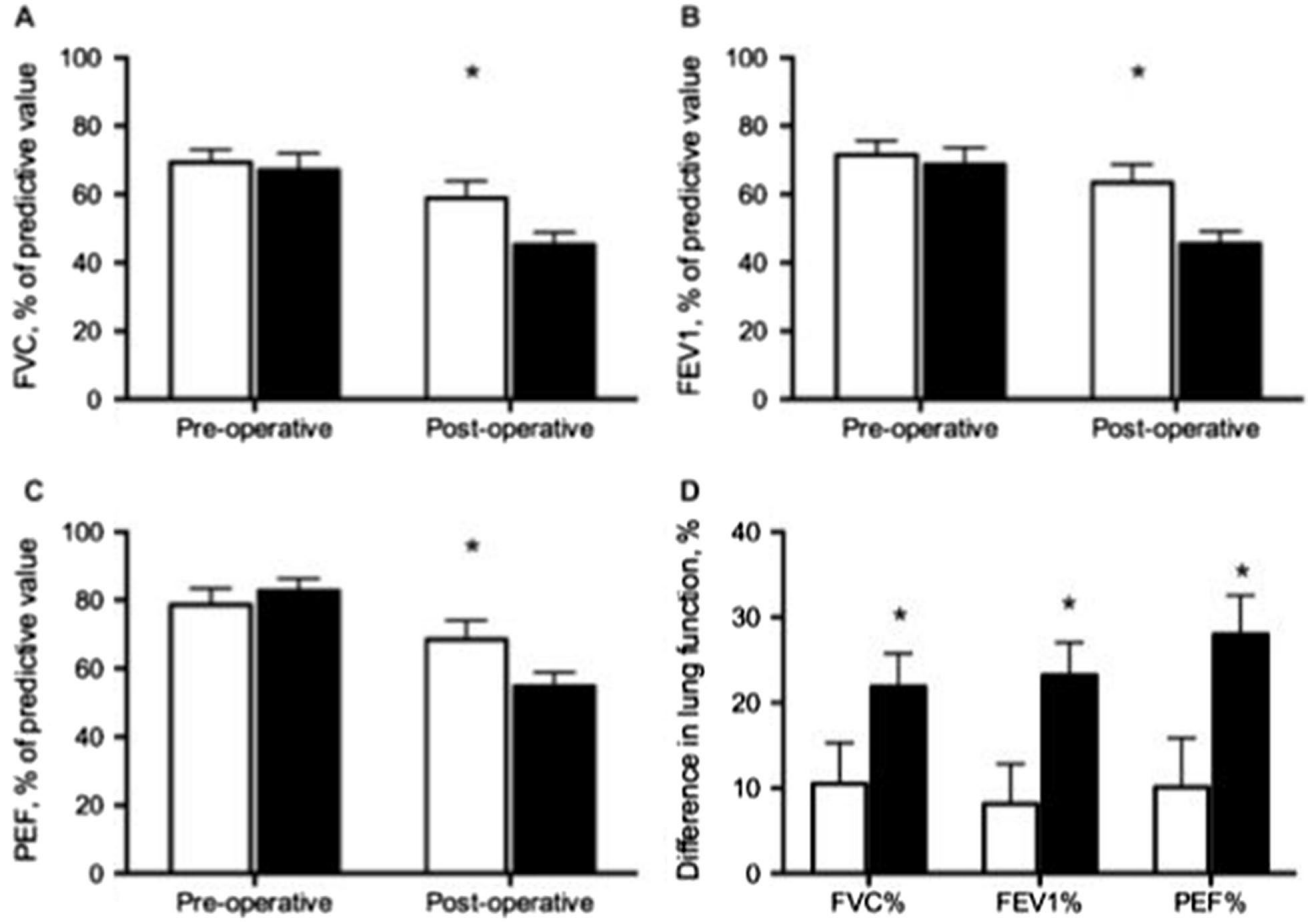
Comparison of lung functions between the subjects who received mechanical insufflation-exsufflation (MI-E) therapy and the subjects who received intermittent positive pressure breathing (IPPB) therapy. **a** Forced vital capacity (FVC) of predictive value were significantly higher in subjects who received MI-E therapy (open bar), compared to the subjects who received IPPB therapy (black bar) (* indicates *p* < 0.05); **b** Forced expiratory volume in one second (FEV_1_) of predictive value were significantly higher in subjects who received MI-E therapy (open bar), compared to the subjects who received IPPB therapy (black bar) (* indicates *p* < 0.05); **c** Peak expiratory flow rate (PEF) of predictive value were no significant difference between both groups; **d** The difference between pre-operative and post-operative values of FVC, FEV_1_, PEF were significantly higher in subjects who received MI-E therapy (open bar), compared to the subjects who received IPPB therapy (black bar) (* indicates *p* < 0.05); (data expressed as mean ± SEM)

The lung function changes in both groups are shown in Fig. 1 and Additional file 1: Table 1. The forced expiratory volume in one second (FEV_1_) and forced vital capacity (FVC) were observed to decrease after surgery, in both groups. The ratio of FEV_1_/ FVC was within the normal range; indicating a restrictive pulmonary defect after surgery. The percentage of predictive value of post-operative FVC (58.4 ± 4.74 vs. 46.0 ± 3.70%, *p* = 0.042), and FEV_1_ (62.4 ± 5.23 vs. 46.8 ± 3.83%, *p* = 0.017) were significantly inferior in the IPPB group, compared to the MI-E group. In addition, the reductions of FVC, FEV_1_, and PEF from preoperative to postoperative were significantly smaller in the MI-E group, compared to the IPPB group (Fig. 1d). Therefore, pulmonary functions were better preserved in the subjects who received post-operative MI-E therapy, compared to those who received IPPB therapy.

### Complications in the two groups

The incidence of post-operative complications including pneumonia, atelectasis and pleural effusion were observed to be similar in both the groups (Additional file 2: Table 2). However, the incidence of chest pain was significantly higher in the MI-E group, compared to the IPPB group (MI-E vs. IPPB group, 61.9% vs. 16.7%; OR, 0.123; 95% C.I., 0.03–0.45; *p* = 0.002). According to the results of this study, the complications were noted in both groups. Only the incidence of chest pain was increased in MI-E group. There was no any complication specific to MI-E or IPPB. After five days of positive pressure therapy, the percentage of patients who showed improvement of atelectasis was similar in both the groups (MI-E vs. IPPB group, 61.9% vs. 50.0%; OR, 0.62; 95% C.I., 0.20–1.91; *p* = 0.40) (Table 2). Although the percentage of improvement of atelectasis in the MI-E group was higher, compared to the IPPB group, in subjects with segmental or lobar atelectasis, the difference was not statistically significant.

**Table 2:** The percentage of improvement in atelectasis after respiratory therapy in post-cardiac surgery patients

Percentage of improvement	MI-E^*^ group	IPPB^†^ group	OR^‡^, 95% C.I.^§^	*p* value
Total atelectasis, N (%)	9/13(69.2)	8/15(53.3)	1.97 (0.42–9.32)	.460
Segmental atelectasis, N (%)	8/8(100)	8/13(61.5)	11.0 (0.52–231)	.111
Lobar atelectasis, N (%)	1/5(20.0)	0(0)	1.67 (0.05–58.3)	.999

MI-E*: Mechanical insufflation-exsufflation, IPPB^†^: intermittent positive pressure breathing, OR^‡^: Odds ratio, C.I. ^§^: Confidence interval

## Discussion

The present study demonstrated that cardiac surgery patients who received MI-E therapy had better lung function compared to those who received IPPB therapy. The incidence of post-operative pulmonary complications including pneumonia, atelectasis, and pleural effusion, were similar in both the groups. However, the number of subjects who experienced chest pain was higher in the MI-E group, compared to the IPPB group.

Compatible with previous reports [14], the current study observed significant decline in the values of effort-dependent lung function tests, such as FVC, FEV_1_, and peak expiratory flow rate, after surgery in both groups. The study subjects developed a proportional decrease in lung volume without change in the FEV_1_/ FVC ratio; indicating a restrictive pulmonary defect after surgery. The normal activity of most of the respiratory muscle groups is impaired after a major surgery, including the airway muscles, abdominal muscles, and diaphragm [14]. Factors contributing to this dysfunction include anesthetic agents and neuromuscular blocking agents, drugs used for post-operative analgesia, pain, disturbed sleep patterns and the inflammatory response to surgery. In addition to the simple muscle weakness, the etiology of decreased lung function may involve poor sputum clearance, poor co-ordination between muscle groups and failure of the normal physiological reflexes, which control the effort-dependent lung function. Theoretically, MI-E therapy may simulate coughing and facilitate better sputum clearance in patients. In the present study, the percentage of improvement of segmental and lobar atelectasis in the MI-E group was observed to be higher, compared to the IPPB group. However, the difference was not statistically significant; probably due to the inadequate sample size. Therefore, further research is mandatory to elucidate the effects of MI-E therapy on the improvement of atelectasis and lung function.

Several studies have demonstrated that pulmonary complications are more common than cardiac complications, in patients undergoing cardiac surgery [2, 5]. In patients who undergo cardiac surgery, the diminution in lung volume provokes atelectasis. Discoid atelectasis is apparently unavoidable, in the immediate postoperative phase. Pleural fluid has been shown to predispose to atelectasis [15]. The high occurrence of pleural effusions and discoid atelectasis leads to their coincidental appearance. In the present study, development of postoperative complications were observed to be similar between the two study groups; indicating that both types of respiratory therapy devices, which were used in the study, are equally effective in preventing post-operative complications. Several devices have been used to reduce post-operative pulmonary complications. IPPB was first introduced in the 1940s [7]. It became particular popular in 1980s and 1990s, before other devices such as CPAP, BiPAP and high-flow nasal cannula therapy became available in clinical practice. However, the role of IPPB is still controversial [6, 16–20]. Literature reports that the incidence of atelectasis in patients who received post-operative IPPB therapy ranges from 40 to 55% [17, 20]. The present study showed that 50% of subjects in the MI-E group and 42.9% of subjects in the IPPB group developed atelectasis, which was concurrent with the previous studies. The incidence of chest pain was significantly higher in the MI-E group, compared to the IPPB group. Due to similar peak airway pressure target and tidal volume setting in both the groups, a probable explanation for the increased incidence of chest pain may be the active negative-pressure exsufflation in MI-E. The MI-E therapy design may simulate a cough or even induce cough and move the secretions towards the mouth. Although the pain could be controlled by remedial medical treatment, caregivers should pay more attention to pain control in cardiac surgery patients, who receive post-operative MI-E therapy.

As is the case with any mechanical positive-pressure device, potential complications of in-exsufflation include abdominal distention, aggravation of gastroesophageal reflux, hemoptysis, chest and abdominal discomfort, acute cardiovascular effects, and pneumothorax. However, rarely have these been reported in literature [11]. Physiological effects on the cardiovascular system were studied during the early phases of development of in-exsufflation. The effects on cardiovascular system include an increase in the peripheral venous pressure (about one third more than during normal coughing), and slight increase in blood pressure [21]. Pulse can increase or decrease with in-exsufflation. Severe bradyarrhythmia has been reported in patients with high spinal cord injury and premature ventricular contractions have been reported in an adolescent with Duchenne muscular dystrophy [21, 22]. Prudent measures to avoid the complications of in-exsufflation include short rest breaks between applications of in-exsufflation; avoid hyperventilation and administration of in-exsufflation before meals or feedings, vigorous medical treatment of gastroesophageal reflux, and adequate treatment of any airway inflammation.

A major limitation of the present study is its retrospective nature, which may have led to bias in the selection of study subjects. Additionally, the sample size of the study is small, and therefore the results should be interpreted with caution. Lastly, a prospective study with a larger sample size is warranted to further confirm the clinical outcomes in patients receiving IPPB or MI-E therapy. Emerging evidence suggests that MI-E therapy is useful in a selected group of patients, such as those with muscle weakness and without airway instability [11]. Nevertheless, before its routine use in clinical practice, more studies are needed to investigate the safety and utility of MI-E therapy in the clearance of secretions from central and peripheral airways, as well as the impact on clinically relevant outcomes, including the incidence of ventilator-associated pneumonia, improvement of atelectasis, length of ICU/ hospital stay, and mortality. If mechanical ventilator day were used as primary aim point, the sample size will be 142 for each group with desired power 80% and α = 0.05.

## Conclusion

Both IPPB and MI-E therapies have similar effects on the prevention of post-operative complications in cardiac surgery patients and both are reasonable choices for post-operative physiotherapy. MI-E therapy showed better improvement in pulmonary functions, but a higher incidence of chest pain, compared to IPPB therapy. However, further studies are required to assess the precise impact of MI-E therapy on post-operative complications.

## Supplementary Information

**Additional file 1.** **Supplement Table 1:** Comparison of post-operative changes in lung functions between the two groups.

**Additional file 2.** **Supplement Table 2:** Comparison of the incidence of post-operative complications between the two groups.

## Data Availability

The data that support the findings of this study are available from The Chang Gung Medical Foundation Institutional Review Board but restrictions apply to the availability of these data, which were used under license for the current study, and so are not publicly available. Data are however available from the authors upon reasonable request and with permission of The Chang Gung Medical Foundation Institutional Review Board.

## Abbreviations

PPCs: Postoperative pulmonary complications
EPCO: European perioperative clinical outcome
FRC: Functional residual capacity
CPAP: Continuous positive airway pressure ventilation
BiPAP: Bilevel positive airway pressure
IPPB: Intermittent positive pressure breathing
MI-E: Mechanical insufflation-exsufflation
ICU: Intensive care unit
PEEP: Positive end-expiratory pressure
FiO_2_: Fraction of inspired oxygen
PEEP: Positive end-expiratory pressure
PFT: Pulmonary function tests
CVS: Cardiovascular surgeon
PA: Postero-anterior
BMI: Body mass index
FEV_1_: Forced expiratory volume in one second
FVC: Forced vital capacity
PEF: Peak expiratory flow
