# Supplementary material for: The effects of mechanical insufflation-exsufflation on lung function and complications in cardiac surgery patients: a pilot study
Source: J Cardiothorac Surg. 2021 Dec 9;16:350. doi: 10.1186/s13019-021-01738-x (PMC8662824; doi:10.1186/s13019-021-01738-x)
Supplement: Supplementary file 1 — Additional file 1. Supplement Table 1: Comparison of post-operative changes in lung functions between the two groups. [file 13019_2021_1738_MOESM1_ESM.docx]

**Supplement Table 1: Comparison of post-operative changes in lung functions between the two groups**

| Variables |  | MI-E^*^ group, n=21 | IPPB^†^ group, n=30 | p value |
| --- | --- | --- | --- | --- |
| FVC^‡^, L | Preoperative | 2.19±0.16 | 2.12±0.18 | .806 |
|  | Postoperative | 1.77±0.20 | 1.39±0.11 | .082 |
|  | Difference | 0.41±0.14 | 0.73±0.13 | .108 |
| FVC^‡^, % | Preoperative | 68.1±3.74 | 67.4±4.85 | .916 |
|  | Postoperative | 58.4±4.74 | 46.0±3.70 | .042 |
|  | Difference | 9.68±4.65 | 21.4±3.61 | .050 |
| FEV_1_^§^, L | Preoperative | 1.78±0.13 | 1.73±0.15 | .804 |
|  | Postoperative | 1.57±0.17 | 1.12±0.08 | .011 |
|  | Difference | 0.21±0.12 | 0.61±0.11 | .017 |
| FEV_1_^§^, % | Preoperative | 69.8±4.35 | 69.4±4.96 | .947 |
|  | Postoperative | 62.4±5.23 | 46.8±3.83 | .017 |
|  | Difference | 7.45±4.49 | 22.6±3.65 | .011 |
| FEV_1_^§^/ FVC^‡^ | Preoperative | 81.0±1.85 | 85.6±1.95 | .106 |
|  | Postoperative | 90.3±4.24 | 81.1±1.59 | .025 |
|  | Difference | -9.32±3.61 | 4.58±1.77 | .001 |
| PEF^\|\|^, L | Preoperative | 76.60±5.12 | 82.9±5.75 | .440 |
|  | Postoperative | 67.1±5.53 | 55.7±4.44 | .111 |
|  | Difference | 9.6±5.52 | 27.2±4.32 | .014 |

MI-E^*^: Mechanical insufflation-exsufflation

IPPB^†^: intermittent positive pressure breathing

FVC: Forced vital capacity

FEV_1_^§^: Forced expiratory volume in one second

PEF^||^: Peak expiratory flow
