# Supplementary material for: The effects of mechanical insufflation-exsufflation on lung function and complications in cardiac surgery patients: a pilot study
Source: J Cardiothorac Surg. 2021 Dec 9;16:350. doi: 10.1186/s13019-021-01738-x (PMC8662824; doi:10.1186/s13019-021-01738-x)
Supplement: Supplementary file 2 — Additional file 2. Supplement Table 2: Comparison of the incidence of post-operative complications between the two groups. [file 13019_2021_1738_MOESM2_ESM.docx]

**Supplement table 2: Comparison of the incidence of post-operative complications between the two groups**

| Variables | MI-E^*^ group, n=21 | IPPB^†^ group, n=30 | OR^§^,  95% C.I. ^‡^ | p value |
| --- | --- | --- | --- | --- |
| Pneumonia | 1 (4.8%) | 2 (6.7%) | 1.43  (0.12-16.86) | .777 |
| Atelectasis | 13 (61.9%) | 15 (50.0%) | 0.62  (0.20-1.91) | .402 |
| -Segmental | 8 (38.1%) | 13 (43.3%) | 1.24  (0.39-3.88) | .709 |
| -Lobar | 5 (23.8%) | 2 (6.7%) | .229  (0.04-1.32) | .099 |
| Pleural effusion | 8 (38.1%) | 14 (46.7%) | 1.42  (0.46-4.43) | .544 |
| Chest pain | 13 (61.9%) | 4 (16.7%) | 0.123  (0.03-0.45) | .002 |

MI-E^*^: Mechanical insufflation-exsufflation

IPPB^†^: Intermittent positive pressure breathing

OR^§^: Odds ratio

C.I. ^‡^: Confidence interval
